# Clinical trials of antihypertensives: Nature of control and design

**DOI:** 10.4103/0253-7613.75659

**Published:** 2011-02

**Authors:** Bhaswat S. Chakraborty

**Affiliations:** Cadila Pharmaceuticals, Ahmedabad, Gujarat - 387 810, India

**Keywords:** Blood pressure, endpoints, good clinical practices, hypertension, randomized clinical trials

## Abstract

This paper reviews the critical issues in the control and design of antihypertension (anti-HT) clinical trials. The international guidelines and current clinical and biostatistical practices were reviewed for relevant clinical, design, end-point assessments and regulatory issues. The results are grouped mainly into ethical, protocol and assessment issues. Ethical issues arise as placebo-controlled trials (PCTs) for HT-lowering agents in patients with moderate to severe HT are undertaken. Patients with organ damage due to HT should not be included in long-term PCT. Active-control trials, however, are suitable for all randomized subsets of patients, including men and women, and different ethnic and age groups. Severity subgroups must be studied separately with consideration to specific study design. Mortality and morbidity outcome studies are not required in anti-HT trials except when significant mortality and cardiovascular morbidity are suspected. Generally, changes in both systolic and diastolic blood pressures (BP) at the end of the dosing interval from the baseline are compared between the active and the control arms as the primary endpoint of anti-HT effect. Onset of the anti-HT effect can be studied as the secondary endpoint. For maintenance of efficacy, long-term studies of ≥6 months need to be undertaken. Error-free measurement of BP is a serious issue as spontaneous changes in BP are large and active drug effect on diastolic BP is often small. Placebo-controlled short-term studies (of ~12 weeks) for dose-response and titration are very useful. Safety studies must be very vigilant on hypotension, orthostatic hypotension and effects on heart. In dose-response studies, at least three doses in addition to placebo should be used to well characterize the benefits and side-effects.

## Introduction

The global burden of hypertension (HT) and comorbid cardiovascular diseases (CVD) is becoming heavier than ever before with each passing year. It is estimated that by 2025, up to 1.58 billion adults worldwide will suffer from some complications of or from HT.[1] That makes one out of each three adults, on an average, will develop clinical HT or its co-morbidities or both. Currently, the prevalence of HT varies around the world, with the lowest prevalence in rural India (3.4% in men and 6.8% in women)[2] and the highest prevalence in Poland (68.9% in men and 72.5% in women).[2] However, in fact, the low-prevalence rates, e.g. as those cited for India, do not necessarily mean a really low occurrence of the disease in this population. Even in those who are diagnosed with HT, treatment is frequently inadequate. In any case, regardless of the prevalence rate, large or small, HT and related diseases must be intervened for prevention, diagnosis and control.

According to the latest JNC, i.e. JNC7, in patients older than 50 years of age, systolic blood pressure (SBP) of >140 mmHg is a more important CVD risk factor than diastolic BP (DBP).[3] However, beginning at 115/75 mmHg, the CVD risk doubles for each increment of 20/10 mmHg. In addition, those who are normotensive will have a 90% lifetime risk of developing HT at the age of 55 years.[3–5] Obesity, dyslipidemia (lower high-density lipoprotein levels), insulin resistance and diabetes mellitus (DM) are the disease conditions that often coexist with HT. Perhaps, physical inactivity and genetic cofactors are involved in these comorbidities. Reduced physical activity is also a risk factor in coronary heart disease (CHD). Other heart conditions such as congestive heart failure (CHF) and fatal or nonfatal myocardial infarctions (MI) and ventricular hypertrophy (VH) also cause elevated BP, and BP-lowering drugs have been found efficacious to different extents in such conditions.[6,7] The direction of discovery of new and appropriate anti-HT interventions ought to be inclusive of those that may even replace the first line of treatment, with superior safety and efficacy profiles. This paper is an attempt to review the important control and design issues involving the higher-phase clinical research of the discovery of new HT drugs.

## Early and Current HT Trials

Randomized clinical trials (RCTs) of anti-HT drugs are mentioned in the literature that date back to the late 1960s to early 1970s. These early trials were carried out in rather select populations (both high- and low-risk elderly patients), and were mainly interested in efficacy measurement of the anti-HT drugs in lowering BP relative to placebo or no treatment. The trials of the 1980s focused on middle-aged hypertensive individuals and later on the elderly. These trials mainly investigated the efficacy of β-blockers and diuretics in reducing the systolic BP in these patients and, subsequently, the DBP.

These early efficacy trials did not address the effect of anti-HT agents in controlling serious comorbidities like MI, CHD and CHF. Their compliance with Good Clinical Practices (GCP) was also rather poor.[8] During the 90s, large randomized trials in a much broader patient population came in vogue and a great portion of these trials elucidated whether and whom to treat with different classes of anti-HT drugs. Continuing to date, these trials of angiotensin converting enzyme inhibitors (ACEIs), β-blockers, angiotensin receptor blockers (ARBs) and calcium channel blockers (CCBs) investigated the exact end-point goals, such as reduction of a certain percent of risk of MIs in Grade II HT patients over 1 year of treatment. The trials evaluated, often, the superiority of the end-point reduction by one agent over another as opposed to the overall efficacy of one agent or the other. Reduction in the relative risk of mortality from the primary and secondary outcome measures is one of the main objectives of the RCTs over the last 5–7 years.[8,9]

The reason for the outcome measurement shifting from lowering of BP to other parameters is that for some drugs, the BP lowering is an inadequate marker (surrogate) of health benefits in HT. Anti-HT drugs can have other important actions that may alter the benefit of lowering of BP. For example, many anti-HT drugs have shown consistent beneficial effects on long-term mortality and morbidity, most clearly on stroke and less consistently on cardiovascular events, such as, low- and high-dose diuretics, reserpine and β-blockers, usually as part of combination therapy (FDA guideline).[10]

## Ethical Considerations

The ethical considerations in HT trials arise mainly on two accounts. Firstly, is it really ethical to put a hypertensive patient on a placebo arm in a RCT or there has to be an active control of some sort in all these trials? Very special populations who are either fragile or cannot make their own decision represent the other main concern for the HT trials.

### 

#### Placebo-controlled HT trials

Ethical considerations in HT trials arise mainly on two accounts. Firstly, is it really ethical to put a BP patient on a placebo arm in a RCT or there has to be an active control of some sort in all these trials? Very special populations who are either fragile or cannot make their own decision represent the other main concern for the HT trials. Placebo-controlled HT clinical trials have been found to be very useful especially when the efficacy of BP lowering is to be measured in an RCT. They have also been found to be useful in the determination of the end points as a direct effect of therapeutic intervention. Despite the availability of a standard and efficacious treatment, the use of placebo-controls in CTs can be considered ethical when withholding the effective treatment leads to no serious adverse events (SAEs) and patients are fully informed about the available therapies and the consequences of delayed treatment.[11] As mentioned above, in case of the HT trials, placebo-controlled efficacy studies are particularly helpful in thoroughly quantifying the effect of treatment. The hazard to patients can be minimized by exposing them to placebo for the least possible time and obtaining proper informed consent from them. In addition, particular attention should be paid to reducing the probability of CV events by excluding subjects with severe HT or major concomitant risk factors.

There are several good reasons why placebo-controls, when appropriate, are preferred to active controls. Firstly, placebo-controlled trials (PCTs) are sensitive in distinguishing an estimate of pure effectiveness of the treatment without any external reference. This kind of estimation, however, is not useful for equivalence, noninferiority and superiority trials. In these later trails, either a clinically meaningful effect of the control has to be preserved or exceeded. In addition, PCTs require a smaller sample size to attain statistical significance than comparing the experimental therapy to another treatment. As a result, these trials are faster and less expensive than active control ones, exposing fewer subjects to the potential risks of the experimental treatment.[12]

## Use of active controls in HT trials

Many major trials for anti-HT drugs that have been conducted in the last 10 years; ALLHAT,[13,14] INVEST,[15] LIFE,[16] LIFE-ISH[17] and RENAAL[18] belong to the category of active control RCTs. Basically, two types of studies have successfully used an active control. The first groups of studies are those in which preservation of a clinically meaningful efficacy and safety margin is essential (equivalence and noninferiority trials, respectively). Examples are INVEST,[15] LIFE[16] and LIFE-ISH[17] studies. In the second group of studies using an active control, the margin of clinically meaningful effect is to be exceeded, as seen in superiority trials. The ALLHAT trial used chlorthalidone, a diuretic, as an active control standard treatment in order to determine the superiority of amlodipine, lisinopril or doxazosin.[13,14]

Usually, active control RCTs are designed in anticipation of CV events in patients at high risk and where long-term effects of the new or experimental interventions are to be observed. These trials, consequently, require, relatively, a higher number of patients in order to achieve the required number of end points rapidly or at the earliest possible time for statistical analysis. Because the effect of experimental treatment separated both from those of active control and natural history with data often requiring appropriate baseline correction, data from these studies are rather complex to analyze.[10]

Both safety and logistical problems can arise from the use of an active control, especially in long-term trials. Such a control can sometimes be very expensive. The long-term trials also require careful term monitoring of SAEs, including irreversible toxicities and deaths in participating patient-subjects. In addition, active control trials, because of their long duration of experimentation, can show cumulative CV toxicities. Such problems must be overcome either through the study design (e.g., dose titration) or through the intervention of an appropriate, but not interfering with experimentation, treatment. If these problems with active controls cannot be overcome at all, development of new products must be abandoned.[10,12]

## Protocol Considerations

### 

#### The trial patient population

For participation in RCTs for HT or its comorbidities, any patient with a BP >120/80 mmHg, especially with one additional risk factor such as body mass index >25, is at risk of developing clinical HT and may qualify for the trial. For example, BP values between 130 and 139/85 and 89 mmHg are associated with a more than two-fold increase in the relative risk from CVD as compared with those with a BP of 120/80 mmHg or below.[3] Patients who are of 55 years of age or more and are also obese or diabetic are particularly risk-prone. A complete list of cardiovascular risk factors is provided in JNC-7.[3] Currently, the patient population studied with a new anti-HT includes a broad range of patients with HT and its comorbidities. For mild to moderate HT, however, only BP can be studied in a CT by measuring both DBP and SBP over the study period. More severe HT is usually studied with relevant concomitant illness, e.g. CHD and DM. Care should be taken that the drugs they need would not interfere with the observations of the effects of the study drug. For example, for patients with CHF, standard treatment requires use of one to several agents (ACE inhibitors) affecting BP, which could have pharmacological actions similar to those of the study drug.[10]

Grading of HT together with target organ damage (TOD) secondary to HT needs to be established accurately. Patients, e.g. with BP >160/110 mmHg and DM, cannot be included in PCTs. Such patients, however, can be included in active-controlled trials with proper safety monitoring. Patients from relevant demographic subsets should be studied, including both men and women, racial/ethnic groups pertinent to the region and both young and older patients. The very old or “fragile elderly,” i.e.. patients >75 years old, should be included. In general, all population subsets should be included in the same studies rather than conducting studies in subgroups. This facilitates comparisons across subsets in the same environment. An exception would be severity of the subgroups, where study designs could be different for different severities. Patients with secondary HT, isolated systolic HT, HT during pregnancy and children with HT should be studied separately if specific indications for use in those populations are being sought.[10]

#### RCTs of antihypertensive agents

All recent trials, since the 1990s, for the assessment of efficacy and safety of anti-HT drugs have been designed and conducted as randomized, blinded trials.[18–22] Such trials are not only free of experimental bias but they are also balanced in all important aspects of the study and differ only in the intervention that the experimental and the control groups receive. As discussed further in the following section, many big trials, such as INVEST and ALLHAT, are designed as prospective, randomized, open, blinded-end point evaluation (PROBE) investigations in thousands of patients in multiple countries. In some studies, there is also an emphasis on the determination of the reduction of mortality and/or CV morbidity by the experimental treatments rather than measuring just the BP-lowering effects.[19,20] Well-designed and well-conducted RCTs have been able to estimate such complex end-points with a great deal of success.

#### Study design and randomization

**Figure 1:**
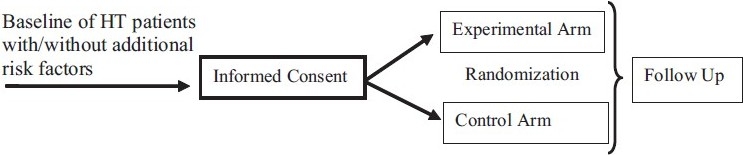
Schematic representation of a two-arm randomized clinical trial of new and existing antihypertension (anti-HT) treatments in HT patients with one or more additional risk factors

Most long-term HT trials are designed today as PROBE studies. Such studies are aimed at comparing a treatment regimen of newer anti-HT drugs (e.g., a CCB) with a traditional regimen (β-blocker and/or a diuretic), like prevention of CHD. The study basically consists of two arms, i.e. the control and the experimental arms, in which appropriate number of patients are entered following a randomization scheme. Because the control arm in such studies consists of receiving an active drug, which is often one of the standard first-line treatments, the trial is often dubbed as an “active control trial,” as mentioned above several times. A schematic representation of a two-arm randomized trial is presented in Figure 1. The total number of patients (sample size) and those in each arm are calculated carefully such that a clinically meaningful effect size can be differentiated between the average outcome measurements for the two arms with adequate statistical power (usually 80%) and significance (usually two-sided 5%). Both the power and the level of significance are prospectively defined and finalized in the detailed protocol before the trial begins. The final sample size includes considerations of drop out patients and all interim analyses (IA)[21]

The RCTs for HT and comorbidities are usually large (few thousand patients) multicentric studies.[19,20] Open label refers to a nonconcealment of both the active control and the experimental drugs to the patients and investigators, in that they can figure out the difference in physical and organoleptic properties between the two. Sometimes, there could also be a difference in the route of the two administrations. The investigator or the expert who measures the endpoint, however, is blinded to the randomization codes and allocations to all patients, such that no bias is introduced in the assessment of the endpoint. If dosage titration for the experimental arm is required, the same principle of endpoint blinding should be applied.[10]

Although there may be many subvarieties, essentially, there are three basic ways to generate a randomization scheme for an RCT. The first approach can be called “simple randomization,” which is equivalent to tossing a coin for each subject that enters a trial. The heads get the experimental treatment while the tails receive the placebo. A computerized or tabulated random number generator is generally used. It is simple and easy to implement and treatment assignment is completely unpredictable. The second approach to randomization, called the “block randomization,” is very popular and balanced within each block. For a trial of *n* treatments, the total number patients are divided into *m* blocks of size 2*n*. Then, each of the *m* blocks is randomized such that *n* patients are allocated to each of the treatments. One can then choose the blocks randomly. The INVEST[15] study followed this scheme of block randomization. Yet, a third approach to randomization involves “stratified blocks.” Because a trial may not be considered valid if it is not well balanced across the prognostic factors, stratification of patients is done to produce comparable groups with regard to certain characteristics (e.g., gender, age, race, disease severity). This approach produces valid statistical tests in all stratified subgroups (e.g., high-risk subgroups in the ALLHAT trial).[13,14]

Whatever the mode of randomization is, it is ensured that the pattern of assignment of control or experimental drug within a group of patients cannot be guessed at any point. It is recommended that the statistician who generated the randomization codes does not get involved in the IA or the final analysis of the experimental data.

Other study designs can be used in HT trials as long as they are scientifically valid and manageable. Placebo controls have been described elsewhere in this article. True double-blinding of patients as well as the investigators is very difficult to achieve as the regimens in the two arms differ on a number of noticeable properties.[10]

Usually, studies are designed for observation and analysis of the primary outcome on which the sample size calculation is also based. Secondary outcomes, however, can also be validly analyzed if the primary outcome difference is not statistically significant provided that they were declared *a priori* and are clinically important. Another condition for the valid use of secondary outcomes in the efficacy or endpoint estimation is that the method to capture outcomes was the same in each treatment group and the data are unbiased (randomized). In addition, if the outcomes for secondary endpoints such as heart failure (HF) and CVD are still compelling even after considering the number of comparisons made, then the conclusion based on these outcomes is valid.

#### Inclusion-exclusion criteria for entry into the study

Following any standard inclusion and exclusion criteria may prove too restrictive or too liberal in a HT clinical study. Inclusion should be based on three basic scientific questions, viz. (a) what is the primary objective of the study?, (b) which clinical symptoms, tests and physical parameters would represent the true patient population? and (c) which clinical symptoms, tests and physical parameters will distinguish the outcome from baseline as well as from control with sensitivity and accuracy? Similarly, the exclusion of all those patients who are likely either refractory to the experimental regimen or marginally meet the true and desirable patient criteria is based on having an experimental sample that will provide for the estimation of a clear and sharp effect size.

The rationale of the inclusion and exclusion criteria for a large, long-term RCT for a new anti-HT agent [Table 1] can be exemplified as follows. Say, the primary endpoint for this trial (comparing a new ARB with an existing combination of β-blocker and diuretic) is reduction of fatal CHD and nonfatal MI. Both men and women of age 55 years or more with SBP and/or DBP ≥140/90 mmHg but ≤180/110 mmHg (treated before or untreated) at two visits with no washout period leading to randomization can be recruited.[3] Addition of one or more risk factors will give a representative sample of patients who are most likely to be benefited from the treatments and also a sensitive and accurate baseline to which the poststudy primary outcome between the two arms can be compared and contrasted. Therefore, patients with at least one additional risk factor should be recruited for this trial and randomly assigned to one of the arms.

**Table 1:** Usual inclusion/exclusion criteria for hypertension randomized clinical trial

*Sr. no.*	*Inclusion criteria*	*Exclusion criteria*
a.	Age/sex: men and women aged > 55 years	Age/sex: men and women aged ≤ 55 years
b.	BP eligibility: Untreated systolic and/or diastolic hypertension (≥ 140/90 mmHg but ≤ 180/110 mm Hg at two visits) No washout period	MI, stroke or angina within 6 months
c.	At least one of the following risk factors: Type 2 diabetes mellitus (DM) HDL cholesterol < 35 mg/dl on any two or more determinations in the past 5 years Left ventricular hypertrophy (past 2 years)	Symptomatic CHF or ejection fraction <35%
	ECG or echo (septum + posterior wall thickness ≥ 25 mm)	
	Current cigarette smoking	
d.		Known renal insufficiency – creatinine ≥ 2 mg/dl
e.		Requiring diuretics, CCB, ACEI or α-blockers for reasons other than hypertension

## Analysis of the Study

### 

#### Evaluation of efficacy

A detailed discussion of the statistical methods employed in analyzing HT trial data is beyond the scope of this paper. However, in general, in efficacy trials, comparison of the mean blood BP lowering can be done by conventional tests of hypothesis, e.g. t-test to compare the relative efficacy of the two treatments. On the other hand, in more complex, long-term end-point trials aiming to examine efficacy as well as reduction of morbidity and mortality, comparison of relative risks or hazards of two treatments is made. In these latter studies, analyses of time to event data and Cox regression are often employed to estimate the relative benefits of treatments.[20]

#### Evaluation of safety

All anti-HT agents have adverse effects on various organs and systems and can also exacerbate a preexisting damage when used chronically. Because a tremendous volume of safety data is generated in the HT-RCTs, maintenance of a database and proper evaluation of the safety profile is required not only during the trial but also at regular intervals post approval. ICH E1 guidance suggests that a database of about 1500 patients (300-600 for 6 months, 100 for 1 year) is usually sufficient for chronically administered drugs. Even larger databases may be required for large trials with commitments of long follow-up periods before and after the approval of the trial drug.[3,20,22]

Attention must be given to the BP-related adverse events in all HT trials, such as excessive hypotension, orthostatic hypotension and rebound phenomena. Depending on the particular drug and other observations, studies of effects on heart rhythm or cardiac conduction, coronary steal effects, effects on risk factors for cardiovascular disease (e.g., blood glucose, lipids) and further deteriorating effects on TOD can also be carried out.[10]

#### Summary

This article deals with control and design principles that guide the conduct and conclusion of a meaningful HT trial. By no means can this replace the blue print of medical documents, standard operating procedures, expertise and organization that are required for a successful trial. Each trial is as meaningful as the number of scientific questions it answers and paves the direction of future trials in that field. Compliance to GCP opens up the data for correct interpretation. Any neglect toward this end of abiding by the GCP principles can raise a slew of critical questions, eventually rendering the entire data to be uninteresting or suspect. Principles of study design and analysis mentioned in this article can give the trialists an advantage of a well-designed study, providing the crucial evidence of safety and efficacy of the agent under testing.
